# Light-induced synchronization of the SCN coupled oscillators and implications for entraining the HPA axis

**DOI:** 10.3389/fendo.2022.960351

**Published:** 2022-10-27

**Authors:** Yannuo Li, Ioannis P. Androulakis

**Affiliations:** ^1^ Chemical & Biochemical Engineering Department, Rutgers University, Piscataway, NJ, United States; ^2^ Biomedical Engineering Department, Rutgers University, Piscataway, NJ, United States

**Keywords:** SCN, circadian, cortisol, HPA, seasonal

## Abstract

The suprachiasmatic nucleus (SCN) synchronizes the physiological rhythms to the external light-dark cycle and tunes the dynamics of circadian rhythms to photoperiod fluctuations. Changes in the neuronal network topologies are suggested to cause adaptation of the SCN in different photoperiods, resulting in the broader phase distribution of neuron activities in long photoperiods (LP) compared to short photoperiods (SP). Regulated by the SCN output, the level of glucocorticoids is elevated in short photoperiod, which is associated with peak disease incidence. The underlying coupling mechanisms of the SCN and the interplay between the SCN and the HPA axis have yet to be fully elucidated. In this work, we propose a mathematical model including a multiple-cellular SCN compartment and the HPA axis to investigate the properties of the circadian timing system under photoperiod changes. Our model predicts that the probability-dependent network is more energy-efficient than the distance-dependent network. Coupling the SCN network by intra-subpopulation and inter-subpopulation forces, we identified the negative correlation between robustness and plasticity of the oscillatory network. The HPA rhythms were predicted to be strongly entrained to the SCN rhythms with a pro-inflammatory high-amplitude glucocorticoid profile under SP. The fast temporal topology switch of the SCN network was predicted to enhance synchronization when the synchronization is not complete. These synchronization and circadian dynamics alterations might govern the seasonal variation of disease incidence and its symptom severity.

## Introduction

Virtually all organisms possess an endogenous time-keeping system that entrains the biological and behavioral rhythms to a circadian 24 *h* period induced by light/dark cycles (*zeitgeber*). In mammals, the circadian clock forms a hierarchically organized network with the primary circadian pacemaker located in the suprachiasmatic nucleus (SCN), which converts environmental photic cues into hormonal, metabolic, and neuronal signals which subsequently synchronize the peripheral clocks and behavioral activities (1–3). The circadian timing system resembles a clock shop rather than a single clock due to the widespread existence of clocks throughout the body (4). As a result, the robust rhythmicity of such a system is indispensable for temporally coordinating organ functions, while the disruption or misalignment of the rhythms is strongly associated with various widespread diseases (5).

Due to the revolution of the earth, physiological adjustments also correspond to seasonal changes to increase survival and reproductive success by relying on signals such as temperature, rainfall patterns, food availability, and duration of daily illumination (6). The latter, which is also defined as photoperiods, has been identified as the most pervasive factor influencing the properties of circadian entrainments, such as phase, amplitude, and degree of synchronization (7). The photoperiod varies across seasons, especially in high latitudes, with photoperiods longer in the summer and shorter in the winter.

Accordingly, the SCN has evolved to adapt to the different photoperiods and exhibits distinct behaviors in long photoperiods (LP) and short photoperiods (SP). The heterogeneous SCN network consists of ~ 20,000 neurons coupled through neurotransmitters. *In vivo*, SCN neurons must synchronize to environmental cycles and each other to generate a coherent circadian output signal to entrain the downstream clocks (8). While rhythms of electrical activity for individual cells remain comparable across seasons, the amplitude of activity at the ensemble level is dependent on the degree of synchronization among neurons (9). Experimental evidence indicates that the degree of synchronization between SCN neurons in mice is lower under LP, and the neuronal phases are more widely distributed (3). Moreover, the peak of the SCN’s output is during the day, and the trough is during the night in both diurnal and nocturnal species (10), and transitions in behavioral patterns between rest and activity occur at half-maximal levels of the SCN’s activity rhythm. Therefore, the increased ensemble amplitude of the SCN in SP due to the narrowing of the neuronal phase distribution results in the physiology and behavior of organisms being shorter during SP than during LP (9, 11). Although much is known about the mechanisms of light entrainment of the circadian timing system, relatively little is known about the internal synchronizing strategies of the SCN (8, 12).

Multiple studies have highlighted that the SCN is typically organized into the ventrolateral “*core*” region (VL) and the dorsomedial “*shell*” region based on anatomical and neurochemical content differences. Experimental evidence suggests that light-induced gene and protein expression occurs mainly in the VL core region in rats (13, 14), hamsters (15), and mice SCN (16), with the precise peptidergic identity of directly retinol-recipient cells species-dependent (17). In rats, the ventrolateral core mainly contains vasoactive intestinal polypeptide (VIP)-expressing neurons, whereas the dorsomedial shell predominantly contains arginine vasopressin (AVP)-expressing neurons. Other neurotransmitters such as gastrin-releasing peptide (GRP) and Neuromedin S (NMS) also exist and contribute to the activity of the SCN (18). Moreover, almost all SCN neurons are γ-amino butyric acid (GABA)-ergic (19). Although the specific mechanisms of different neurotransmitters might be different, those neurotransmitters are speculated to regulate neurons through a mutual coupling mechanism (20, 21), where they are released in a circadian fashion and exhibit feedback on the clock genes. The SCN’s function is dependent on the collective activity of the neurons and the inter-neuronal coupling (12, 22). Evidence for the SCN heterogeneity has led to various mathematical models of its network (21, 23, 24). Thus, several common network topologies have been examined in modeling the SCN’s structure, including all-to-all connectivity networks, regularly connected networks, Newman-Watts (NW) small-world networks, and scale-free networks (25). These models, while varying considerably in their complexity and underlying assumptions, all converge on the inclusion of a population of heterogeneous oscillators coupled with inter-neuronal communications.

Theoretical works suggest that the response of the SCN to photoperiod changes can be effectively regulated by the number of connections between the core and the shell (2, 9, 26, 27). However, due to experimental limitations, the core-shell coupling nature remains unclear (28). The SCN consists of both distance-dependent and probability-dependent connections (2, 29). Specifically, the SCN rhythms of VIP knockout mice were restored by the coculture of WT SCN, implying that small diffusible (distance-dependent) neuropeptides secreted from the WT SCN are critical for the rhythm synchronization (30). On the other hand, optogenetic-assisted circuit mapping observed functional long-range connectivity between VIP hypothalamic neurons and AVP hypothalamic neurons (31), implying the importance of the neuronal network circuitry mediated by probability-dependent synaptic interactions. The above indicates that both the distance-dependent coupling and probability-dependent coupling can contribute to the mechanisms behind the core-shell communication, which are therefore both considered by the present work.

Regulated by the SCN, glucocorticoid (cortisol in humans, corticosterone in rodents) levels secreted by the HPA axis also exhibit significant seasonal rhythmicity, with levels peaking during SP months and reduced during the LP months (32). The humoral glucocorticoid signals synchronize the expression of peripheral molecular clock components, which in turn regulate biological processes including immune function (33), glucose homeostasis (34), and steroidogenesis (35). Abnormal glucocorticoid circadian patterns are associated with chronic conditions such as rheumatoid arthritis (RA) (36), type 2 diabetes (37), metabolic disorders (38), and behavioral disorders such as post-traumatic stress disorder and depression (39). Therefore, the HPA axis plays a central role in response to the photoperiod variations transduced by the SCN, enabling the host to actively re-establish homeostasis in different seasons (40).

Understanding the dynamics of circadian rhythms at a systematic level motivates the need for characterizing the SCN network, interactions between the SCN and the HPA axis, and synchronization variations of the system as photoperiods change. The present study investigates how SCN network topology influences system behavior and circadian dynamics by incorporating a precise single-cell oscillator model into a heterogeneous network of linked oscillators with two possible coupling mechanisms (distance-dependent and probability-dependent) across the core and the shell. By quantifying the synchronization efficiency of two alternative coupling mechanisms, our findings identify the probability-dependent core-shell coupling network as an “ideal” design capable of producing coherent signals with a reduced wiring cost. We further investigated the SCN and HPA axis response under different photoperiod schedules. Our results show that (1) neuronal communications induce systemic oscillations within the SCN even without an external *zeitgeber*; (2) the robustness and plasticity of the oscillatory network are negatively correlated (3) HPA rhythms were strongly entrained to the seasonally varying SCN rhythms with distinct day-length differences; Specifically, our results suggest that the pro-inflammatory high-amplitude glucocorticoids profile occurs during the shorter winter days, which is associated with enhanced immune responses and disease activities; (4) the temporal topology switch of the SCN network increases synchronization when the synchronization is not complete. Thus, our work sheds light on the adaptation strategies of the circadian timing system by examining complicated physiological entrainment structures between the SCN and the HPA axis under different photoperiods.

## Materials and methods

### Model structure

**Figure 1:**
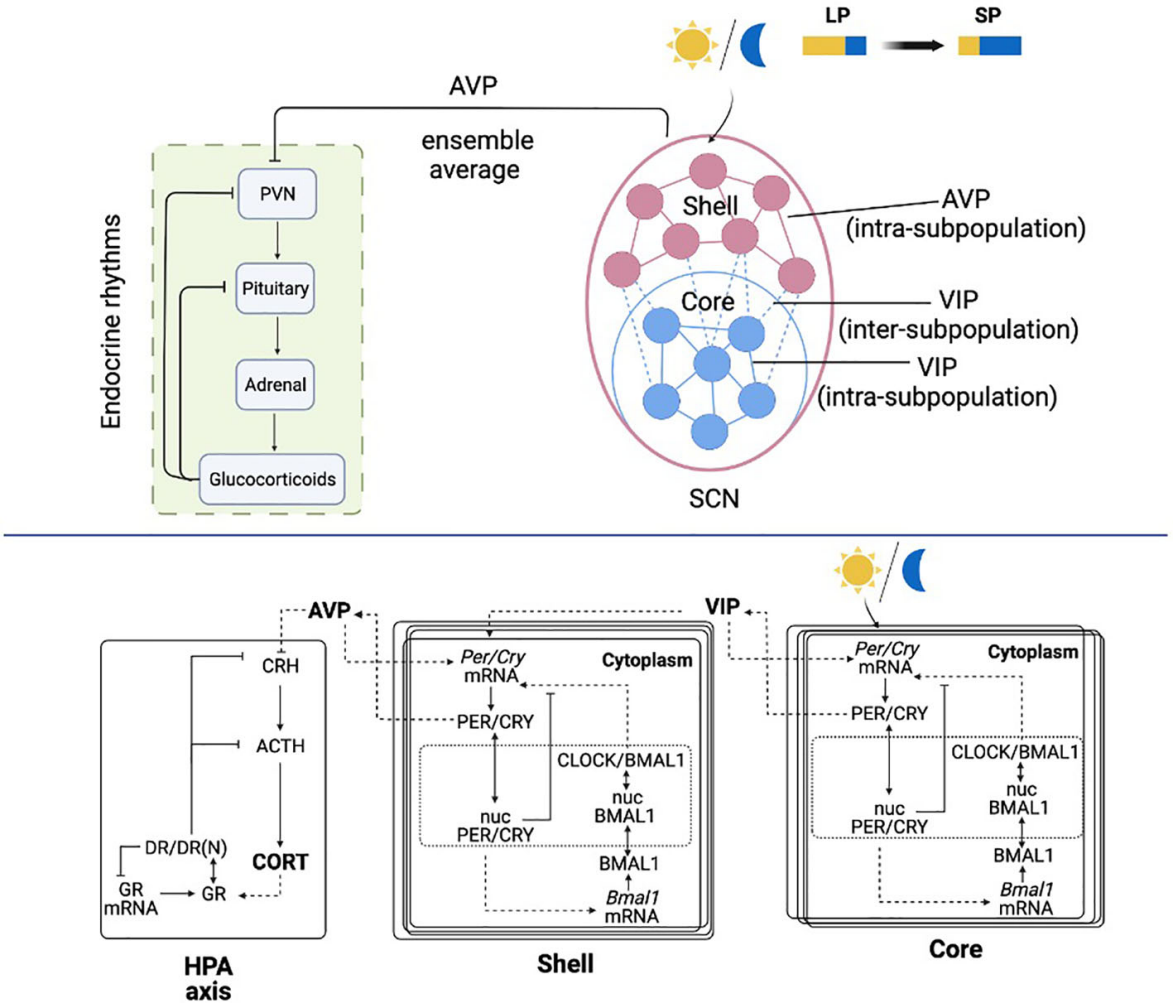
Schematic representation of the light entrained multiple-cellular SCN and HPA axis model. The SCN core is entrained to the light/dark cycle, and the shell is entrained by the core through inter-subpopulation VIP signals. The HPA axis is further entrained by the AVP ensemble average signal from the SCN core.

To mimic the organization of the circadian timing system that consists of the SCN and the HPA axis, our system is mathematically constructed with three compartments: the SCN core, the SCN shell, and the HPA axis (**Figure 1**). The core and the shell are represented as two ensembles of molecular circadian oscillators. At a single-cell level, a detailed molecular model was used to describe the intracellular activities of clock genes and proteins, along the lines of our earlier work (41, 42). At the ensemble level, the cells are coupled by the released neurotransmitters. The HPA axis consists of a single cell model which incorporates the glucocorticoid dynamics as a synchronization outcome of the neurotransmitter AVP secreted from the shell (43). The schematic of the model is shown in **Figure 1**.

The detailed molecular model of the SCN includes a positive and negative feedback loop, which leads to an autonomous oscillation (44). In the positive feedback loop, the PER and CRY proteins translocate into the nucleus (Equations 4 and 12) following their expression (Equations 3 and 11) and activate *Bmal1* mRNA transcription (Equations 5 and 13). After the translation of BMAL1 protein (Equations 6 and 14) and its translocation to the nucleus (Equations 7 and 15), PER/CRY protein indirectly increases the expression of CLOCK/BMAL1 heterodimer (Equations 8 and 16). In the negative feedback loop, nucleus PER/CRY protein inhibits their own mRNA’s transcription by binding to the E box enhancer of the CLOCK/BMAL1 heterocomplex (Equations 2 and 10). As included in **Supplementary Table 1**, the parameters for the core and the shell are adapted from our previous work (45), the suffix “m” denotes parameters in the core compartment, and the suffix “e” denotes parameters in the shell compartment.

In the SCN, neurons communicate with each other *via* the neurotransmitters (46). The neurotransmitters VIP and AVP release is activated by cytosolic PER/CRY proteins in the core and the shell (21, 47) (Equations 9 and 17). The model includes two types of inter-neuronal couplings: (a) intra-subpopulation coupling, which includes the communication within the core and the shell; (b) inter-subpopulation coupling, which includes the coupling signals from the core to the shell. The intra-subpopulation coupling is accomplished by the neurotransmitter secreted within its subpopulation (i.e., VIP for the core and AVP for the shell). The inter-subpopulation coupling is achieved by VIP signals secreted from the core. Both neurotransmitters are hypothesized to induce activation effects on *Per/Cry* mRNA transcription (Equations 2 and 10). The intra-subpopulation coupling is modeled as a constitutive activator term (21, 48). The inter-subpopulation coupling is introduced by an additional term in Equation 10, which accounts for an independent neurotransmitter-driven transcription process apart from the CLOCK/BMAL1 driven transcription (49). Light entrainment of the core is modeled by an additional term in Equation 2 to describe the independent photic activation effects on *Per/Cry* mRNA transcription (45). Although light signals are mainly transduced by the core, it is strongly speculated that the core is not the only light-responsive subregion. Studies have shown that the entire mouse SCN receives dense innervation from retinal ganglion cells (50). However, due to the dense retinal innervation pattern, deciphering the neuron identity innervated by retinal ganglion cells has been difficult, and little is known about which cell types in the SCN receive the retinal input (51, 52). Considering the focus of the present study, we modeled the light transduction pathway in a conventional manner, where the core receives the optic input, and the shell receives input from the core (2, 53).

The model of the HPA axis is adapted from our prior works (43, 45, 54), where its entraining effects are achieved as AVP’s (ensemble average secreted from the SCN shell, Equation 18) down-regulation on the CRH secretion (Equation 19). CRH further regulates the release of ACTH (Equation 20) and eventually induces glucocorticoid (CORT) secretion (corticosterone in this work, Equation 21). Following the transcription and translation of glucocorticoid receptors (Equations 22-23), CORT binds to its receptor (GR) in the cytoplasm of target cells, forming the receptor/CORT complex (DR) (Equation 24). DR subsequently translocates to the nucleus, resulting in the formation of the nuclear-activated receptor, glucocorticoid complex (DR(N)) (Equation 25). DR(N) exhibits receptor-mediated inhibition on CRH and ACTH (Equations 19 and 20) and accounts for the negative feedback loop of the HPA axis. Admittedly, the current HPA model does not provide a complete representation of all the underlying dynamics regulating seasonal changes in the circadian timing system. For example, seasonal photoperiod acts on the pineal gland to secret different levels of melatonin, resulting in seasonal changes in the HPA axis receptor, which may be the pathophysiological basis for the onset of seasonal affective disorder (55). Moreover, the ultradian oscillation of the HPA axis is neglected (56). However, considering the focus of the present work, our model will exclusively investigate the circadian interaction between the SCN and the HPA axis. Studies have shown that many essential properties of glucocorticoid rhythms can be explained using circadian limit cycle oscillators of the HPA axis (39, 57). Therefore, we expect the critical features of our simulation results to be preserved when incorporating the system with more complex components.


(1) $$light=\{\begin{array}{c} 1,\;\;0\leq ZT<PP\\ \;\;0,\;\;PP\leq ZT<24 \end{array}(\text{ZT}:\text{zeitgeber time})$$

Single cell model in the core:


(2) $$\frac{dPer/Cry_{mRNA}{\left(core\right)}_{i}}{dt}=\frac{v_{1bm}\left(CLOCK/BMAL1{\left(core\right)}_{i}+v_{c1}.VIP_{i,intra}^{cm}\right)}{k_{1bm}\left(1\;+{\left(\frac{nucPER/CRY{\left(core\right)}_{i}}{k_{1im}}\right)}^{pm}+CLOCK/BMAL1{\left(core\right)}_{i}+v_{c1}.VIP_{i,intra}^{cm}\right)}-k_{1dm}.Per/Cry_{mRNA}{\left(core\right)}_{i}+v_{l}\frac{light}{light+K_{l}}$$

(3) $$\frac{dPER/CRY{\left(core\right)}_{i}}{dt}=k_{2bm}\cdot Per/Cry_{mRNA}{\left(core\right)}_{i}^{qm}-k_{2dm}\cdot PER/CRY{\left(core\right)}_{i}-k_{2tm}\cdot PER/CRY{\left(core\right)}_{i}+k_{3tm}\cdot nucPER/CRY{\left(core\right)}_{i}$$

(4) $$\frac{dnucPER/CRY{\left(core\right)}_{i}}{dt}=k_{2tm}\cdot PER/CRY{\left(core\right)}_{i}\;-k_{3tm}\cdot nucPER/CRY{\left(core\right)}_{i}-k_{3dm}\cdot nucPER/CRY{\left(core\right)}_{i}$$

(5) $$\frac{dBmal1_{mRNA}{\left(core\right)}_{i}}{dt}=\frac{\nu_{4bm}\cdot nucPER/CRY{\left(core\right)}_{i}^{rm}}{k_{4bm}+nucPER/CRY{\left(core\right)}_{i}^{rm}}-k_{4dm}\cdot Bmal1_{mRNA}{\left(core\right)}_{i}$$

(6) $$\frac{dBMAL1{\left(core\right)}_{i}}{dt}=k_{5bm}\cdot Bmal1_{mRNA}{\left(core\right)}_{i}-k_{5dm}\cdot BMAL1{\left(core\right)}_{i}-k_{5tm}\cdot BMAL1{\left(core\right)}_{i}+k_{6tm}\cdot nucBMAL1{\left(core\right)}_{i}$$

(7) $$\frac{dnucBMAL1{\left(core\right)}_{i}}{dt}=k_{5tm}\cdot BMAL1{\left(core\right)}_{i}-k_{6tm}\cdot nucBMAL1{\left(core\right)}_{i}-k_{6dm}\cdot nucBMAL1{\left(core\right)}_{i}+k_{7pm}\cdot CLOCK/BMAL1{\left(core\right)}_{i}-k_{6pm}\cdot nucBMAL1{\left(core\right)}_{i}$$

(8) $$\frac{dCLOCK/BMAL1{\left(core\right)}_{i}}{dt}=k_{6pm}\cdot nucBMAL1{\left(core\right)}_{i}-k_{7pm}\cdot CLOCK/BMAL1{\left(core\right)}_{i}-k_{7dm}\cdot CLOCK/BMAL1{\left(core\right)}_{i}$$

(9) $$\frac{dVIP_{i,\;\;secreted}}{dt}=k_{vs1}\cdot PER/CRY_{mRNA}{\left(core\right)}_{i}-k_{dv1}\cdot VIP_{i,secreted}$$

Single cell model in the shell:


(10) $$\frac{dPer/Cry_{mRNA}{\left(shell\right)}_{i}}{dt}=\frac{v_{1be}\left(CLOCK/BMAL1{\left(shell\right)}_{i}+v_{c2}.AVP_{i,intra}^{ce}\right)}{k_{1be}\left(1\;+{\left(\frac{nucPER/CRY{\left(shell\right)}_{i}}{k_{1ie}}\right)}^{pe}+CLOCK/BMAL1{\left(shell\right)}_{i}+v_{c2}.AVP_{i,intra}^{ce}\right)}-k_{1de}.Per/Cry_{mRNA}{\left(shell\right)}_{i}+k_{c}.VIP_{i,inter}$$

(11) $$\frac{dPER/CRY{\left(shell\right)}_{i}}{dt}=k_{2be}\cdot Per/Cry_{mRNA}{\left(shell\right)}_{i}^{qe}-k_{2de}\cdot PER/CRY{\left(shell\right)}_{i}-k_{2te}\cdot PER/CRY{\left(shell\right)}_{i}+k_{3te}\cdot nucPER/CRY{\left(shell\right)}_{i}$$

(12) $$\frac{dnucPER/CRY{\left(shell\right)}_{i}}{dt}=k_{2te}\cdot PER/CRY{\left(shell\right)}_{i}\;-k_{3te}\cdot nucPER/CRY{\left(shell\right)}_{i}-k_{3de}\cdot nucPER/CRY{\left(shell\right)}_{i}$$

(13) $$\frac{dBmal1_{mRNA}{\left(shell\right)}_{i}}{dt}=\frac{\nu_{4be}\cdot nucPER/CRY{\left(shell\right)}_{i}^{re}}{k_{4be}+nucPER/CRY{\left(shell\right)}_{i}^{re}}-k_{4de}\cdot Bmal1_{mRNA}{\left(shell\right)}_{i}$$

(14) $$\frac{dBMAL1{\left(shell\right)}_{i}}{dt}=k_{5be}\cdot Bmal1_{mRNA}{\left(shell\right)}_{i}-k_{5de}\cdot BMAL1{\left(shell\right)}_{i}-k_{5te}\cdot BMAL1{\left(shell\right)}_{i}+k_{6te}\cdot nucBMAL1{\left(shell\right)}_{i}$$

(15) $$\frac{dnucBMAL1{\left(shell\right)}_{i}}{dt}=k_{5te}\cdot BMAL1{\left(shell\right)}_{i}-k_{6te}\cdot nucBMAL1{\left(shell\right)}_{i}-k_{6de}\cdot nucBMAL1{\left(shell\right)}_{i}+k_{7pe}\cdot CLOCK/BMAL1{\left(shell\right)}_{i}-k_{6pe}\cdot nucBMAL1{\left(shell\right)}_{i}$$

(16) $$\frac{dCLOCK/BMAL1{\left(shell\right)}_{i}}{dt}=k_{6pe}\cdot nucBMAL1{\left(shell\right)}_{i}-k_{7pe}\cdot CLOCK/BMAL1{\left(shell\right)}_{i}-k_{7de}\cdot CLOCK/BMAL1{\left(shell\right)}_{i}$$

(17) $$\frac{dAVP_{i,secreted}}{dt}=k_{vs2}\cdot PER/CRY{\left(shell\right)}_{i}-k_{dv2}\cdot AVP_{i,secreted}$$

(18) $$AVP(ensemble)=\frac{1}{3N}\sum_{i=1}^{3N}AVP_{i,secreted}\;$$

HPA axis:


(19) $$\frac{dCRH}{dt}=\frac{k_{p1}K_{p1}}{K_{p1}+DR(N)}-V_{d1}.\frac{CRH}{K_{d1}+CRH}.\left(1+\;v_{coe}.\left(1+AVP(ensemble)\right)\right)\;$$

(20) $$\frac{dACTH}{dt}=\frac{k_{p2}K_{p2}CRH}{K_{p2}+DR(N)}-V_{d2}\frac{ACTH}{K_{d2}+ACTH}\;\;$$

(21) $$\frac{dCORT}{dt}=k_{p3}.ACTH/(K_{p3}+ACTH)-V_{d3}\frac{CORT}{K_{d3}+CORT}\;$$

Glucocorticoid receptor pharmacodynamics


(22) $$\frac{dGR_{mRNA}}{dt}=k_{sym,GRm}.\left(1-\frac{DR(N)}{IC_{50,GRm}+DR(N)}\right)-k_{sym}.GR_{mRNA}\;$$

(23) $$\frac{dGR}{dt}=k_{sym,GR}.\;GR_{mRNA}+r_{f}.k_{re}.DR(N)-k_{on}.(CORT).GR-k_{deg,GR}.GR$$

(24) $$\frac{dDR}{dt}=k_{on}.(CORT).GR-k_{T}.DR\;\;$$

(25) $$\frac{dDR(N)}{dt}=k_{T}.DR-r_{f}.k_{re}.DR(N)$$

### Heterogeneous ensembles generation

The SCN consists of a heterogeneous ensemble of cells whose free-running periods and phases vary significantly (58). To account for the heterogeneity of the SCN, we simulated cell-cell variability using Sobol sampling (± 3*%* ) of the parameters of Equations 2-17, which reflect the stochastic expression of intracellular activities. While alternative approaches such as the Gillespie or Chemical Langevin equations exist (41, 42, 59), in the present study, we chose parameter sampling as a simple method to generate a distribution of heterogeneous cells. The nominal parameter sets for the core, the shell, and the HPA axis are adopted from our previous work (45), with their values and descriptions included in **Supplementary Table 1**.

To simulate the experimentally observed SCN topology (47, 60), we randomly distributed the heterogeneous core and shell populations in a lobe-shaped area where the shell surrounds the core. The light-receptive core region contains approximately 25% of the neurons in the SCN, and the light-insensitive shell region processes the remaining 75% of the neurons (61). Consistent with such a ratio, we created *N* and 3*N* neurons for the core and the shell, respectively, as *N* represents the total number of neurons in the core. We acknowledge that the two-dimensional geometry and the distinction of core/shell region are oversimplified since the distribution of three-dimensional SCN neurons shows a more complex spatial organization (62, 63). However, since our study is a first attempt to simulate the interplay between the coupled SCN network and the HPA axis, we aim at a manageable representation of the SCN compartment. Moreover, this simplification strategy was also suggested by several previous SCN models (9, 21, 27).

#### The SCN topology

To investigate the effects of coupling topologies on SCN synchronization, both the distance-dependent and probability-dependent networks have been modeled.

For the distance-dependent coupling model, all the neuronal connections (including core-core, shell-shell, and core-shell) are diffusible, and neurons receive coupling signals depending on their location. The network topology is defined by a binary coupling term *A*_*ij*_ . If there is a directed link between neuronal node *j* and *i* , *A*_*ij*_=1 ; otherwise, *A*_*ij*_=0 . We define *A*_*ij*_=1 if the physical distance between nodes *i* and *j* is smaller than a predefined threshold distance *d* , while *A*_*ij*_=0 if the distance is larger than *d* .

For the probability-dependent coupling model, the intra-subpopulation connections (core-core and shell-shell) are described as distance-dependent, as we assume the released neurotransmitters to have a more significant effect on adjacent cells within the core and shell subpopulations. The inter-subpopulation connections (core-shell), on the other hand, are determined based on a probability *P*_*PT*_ which describes the likelihood that two nodes are connected. To capture the stochastic feature of the core-shell coupling, we consider randomly changing inter-subpopulation topologies during individual simulations. We define the interval under which the topology changes as a “switching period”. For instance, a 12 *h* switching period means that the core and shell connections are randomly re-organized every twelve hours.

Intra-subpopulation neurotransmitter signals received by neuron *i* were computed using Equations 26-29. For simplicity, we only consider unidirectional coupling for all the core-shell connections, following the experimental findings that the core projects densely to the shell while the shell projects sparsely to the core (64).

The inter-subpopulation couplings sensed by each shell neuron are described in Equation 30. To model the experimentally observed differences in synchronization degree of the SCN neuronal oscillators under LP and SP, we set the threshold distance across the core and the shell (*D*_*PT*_ , PT stands for photoperiod) in the diffusible coupling model and the probability (*P*_*PT*_ ) in the probability-dependent coupling model as two tunable parameters dependent on photoperiod. Equations 32 and 34 link the two parameters to the photoperiod changes. As *D*_*PT*_ or *P*_*PT*_ rises with decreasing photoperiod, more core-shell connections will exist during the short photoperiod. It should be noted that while the results of two models run in different seasons will not be quantitatively identical due to the way the model equations were constructed, the overall trends of changes in synchronization degree, phase, and amplitude predicted by the two models are qualitatively similar. Accordingly, the binary coupling terms in the distance-dependent and probability-dependent models are calculated by Equation 31 and Equation 33, separately. The sum of local concentration of neurotransmitters is scaled using the factor $\sum_{j=1}^{N(core)}A_{i,j}$ , which represents the number of signals received by neuron *i* . *N* and 3*N* describe the total cell numbers in the SCN core and shell sub-populations. *d*_*core*_ and *d*_*shell*_ are constants defined as the threshold distances inside the core and the shell.


(26) $$VIP_{i,intra}=\frac{\sum_{j=1}^{N}A_{i,j}*VIP_{j,secreted}}{\sum_{j=1}^{N}A_{i,j}}$$

(27) $$A_{i,j}=\{\begin{array}{c} 1,\;\;\sqrt{{\left(x_{i}-x_{j}\right)}^{2}+{\left(y_{i}-y_{j}\right)}^{2}}<d(core)\\ 0,\;\;\sqrt{{\left(x_{i}-x_{j}\right)}^{2}+{\left(y_{i}-y_{j}\right)}^{2}}\geq d(core) \end{array}$$

(28) $$AVP_{i,intra}=\frac{\sum_{j=1}^{3N}A_{i,j}*AVP_{j,secreted}}{\sum_{j=1}^{3N}A_{i,j}}$$

(29) $$A_{i,j}=\{\begin{array}{c} 1,\;\;\sqrt{{\left(x_{i}-x_{j}\right)}^{2}+{\left(y_{i}-y_{j}\right)}^{2}}<d(shell)\\ 0,\;\;\sqrt{{\left(x_{i}-x_{j}\right)}^{2}+{\left(y_{i}-y_{j}\right)}^{2}}\geq d(shell) \end{array}$$

(30) $$VIP_{i,inter}=\frac{\sum_{j=1}^{N(core)}A_{i,j}*VIP_{j,secreted}}{\sum_{j=1}^{N(core)}A_{i,j}}$$

Distance-dependent network:


(31) $$A_{i,j}=\{\begin{array}{c} 1,\;\;\sqrt{{\left(x_{i}-x_{j}\right)}^{2}+{\left(y_{i}-y_{j}\right)}^{2}}<D_{PT}\\ 0,\;\;\sqrt{{\left(x_{i}-x_{j}\right)}^{2}+{\left(y_{i}-y_{j}\right)}^{2}}\geq D_{PT} \end{array}$$

(32) $$D_{PT}=D_{max}*\frac{S_{distance}}{Photoperiod-K_{distance}}$$

Probability-dependent network:


(33) $$A_{i,j}=\{\begin{array}{c} 1,\;\;probability=P_{PT}\\ 0,\;\;probability=(1-P_{PT}) \end{array}$$

(34) $$P_{PT}={\left(\frac{PP_{max}}{Photoperiod}\right)}^{n_{probability}}*P_{min}$$

#### Quantification of synchronicity and periodicity

In the SCN, the synchronization of heterogeneous oscillations through inter-neuronal communication is essential for generating a consistent physiological rhythm (8). To study how the SCN topology and photic inputs affect the state of the SCN neurons, we evaluate the degree of synchronization among the SCN neurons. We incorporated an order parameter *R*_*syn*_ for *Per/Cry* mRNA, representing the ratio of the mean-field variance over the mean-variance of each oscillator (65).


(35) $$R_{syn,k}=\frac{\langle {\bar{y}}_{k}^{2}\rangle -{\langle {\bar{y}}_{k}\rangle }^{2}}{\frac{1}{n}.\sum_{j=1}^{n}\left(\langle {\text{y}}_{k,j}^{2}\rangle -{\langle y_{k,j}\rangle }^{2}\right)}$$

(36) $${\bar{y}}_{k}=\frac{1}{n}.\sum_{j=1}^{n}{\bar{y}}_{k,j}$$

(37) $$<y_{k,j}>=\frac{1}{T}.\int_{t=t1}^{t=t1+T}y_{k,j}$$

In Equation 35, *y*_*kj*_ is the time vector for cell *j* , component *k* , *n* is the total cell number (*n*=*N* for the core, *n*=3*N* for the shell), ${\bar{y}}_{k}$ is the average time vector across a population of *n* cells, while <*y*_*k*,*j*_> represents the temporal averages of the oscillator.

All *R*_*syn*_ values in the present work have been estimated over a 50-day period once the system reaches a steady-state. When the cells are entirely desynchronized, *R*_*syn*_  has a value of zero, and when the cells are fully synchronized, *R*_*syn*_ has a value of one (24).

The peak time is used to identify the period and phases. We calculated the mean time gap between two consecutive peaks to estimate the period and the time gap between the peak time at steady-state and 12 am (reference phase) to estimate the phase. The phase on angular coordinates was computed using the formula Φ=(2*π*.Δ*t*)/*T* , where Δ*t* is the time lag between the peak and 12 am and T is the period of the calculated component.

## Results

### Comparisons of the synchronization efficiency between distance-dependent and probability-dependent structures

**Figure 2:**
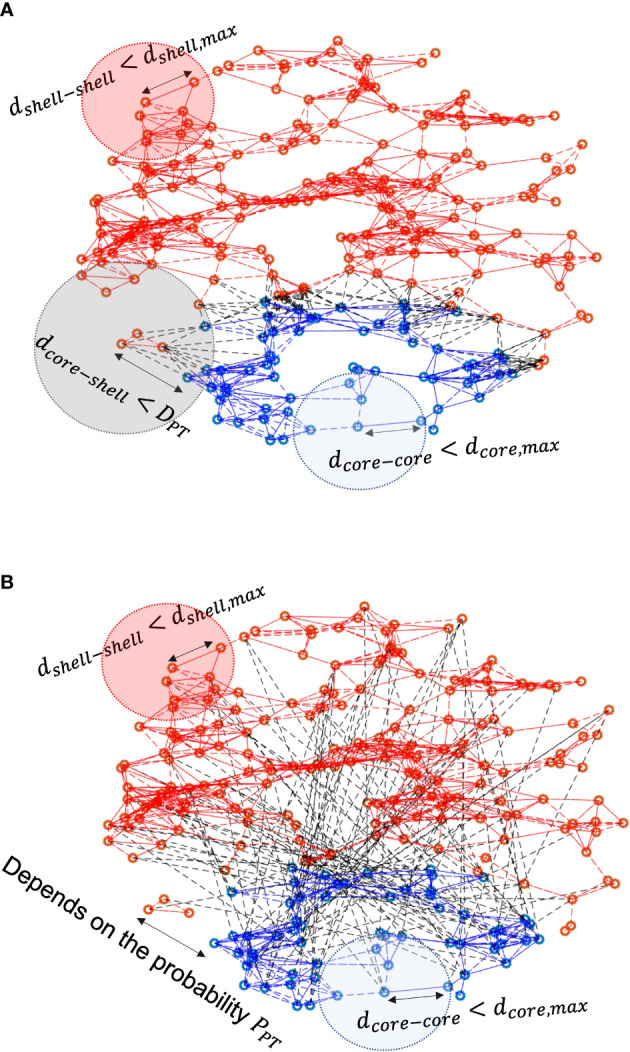
Schematics of **(A)** distance-dependent coupling and **(B)** probability-dependent coupling models. Blue dots refer to neurons in the core, and red dots refer to neurons in the shell. Red/blue lines represent intra-subpopulation couplings within the core and the shell, while black dashed lines characterize inter-subpopulation couplings between the core and the shell. In the distance-dependent model, neurons in the shell sense signals from the core neurons within the threshold distance. In the probability-dependent network, neurons in the shell receive signals from the shell randomly depending on the probability.

The network schematics of distance-dependent and probability-dependent networks are represented in **Figures 2A, B**, respectively. Within the core and the shell, the existence of intra-subpopulation couplings depends on the physical distance between two neurons, as the blue and red dashed lines denote, respectively. In the distance-dependent network, neurons in the shell receive VIP signals only if they are within the threshold of the core. While in the probability-dependent network, the inter-subpopulation couplings are randomly distributed across the core and the shell.

**Figure 3:**
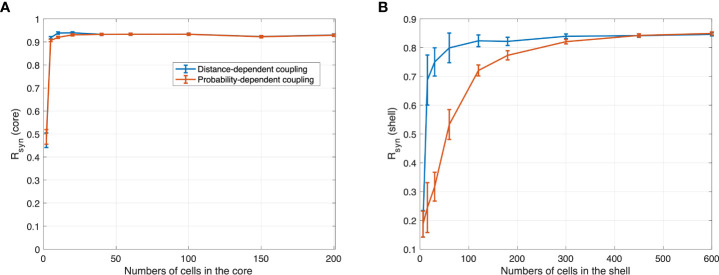
R synchronization values obtained with different numbers of cells in **(A)** core and **(B)** shell under 12 h light 12 h dark for the distance-dependent network and probability-dependent network computed with normalized distance threshold $\frac{D_{PT}}{D_{PT,max}}$ =0.8 and p=0.1. For random networks, each mean *R*_*syn*_ and its standard deviations were computed from the 50 network realizations. The synchronization level in the core increases abruptly as the number of cells increases. Compared to the core, the synchronization level in the shell increases gradually, with the distance-dependent coupling network being more sensitive to the population size.

Before proceeding with the model predictions, it is critical to understand the role the number of neurons in each SCN compartment plays. To better describe the influence of population size, we first analyzed the synchronization at the SCN as a function of population size in both the core and shell. For small population sizes, the degree of synchronization was significantly dependent on the number of cells in both the core and the shell, as shown in **Figure 3**. Based on our model, cell entrainment is dependent on the entraining signal as well as the cell-cell communications. Since the core is directly entrained by the *zeitgeber*, the number of cells plays a weaker role. As such, a very small critical number of core neurons of about *N*≈20 cells is enough to produce an entrained population of cells. The picture in the shell is somewhat more complicated. Shell neurons receive the weaker in strength, core-produced VIP signal which acts as a “global” entrainer, as well as signals from other shell neurons. We observe, in **Figure 3** (right), that *R*_*syn*_ is dependent on the nature of core-shell communication. Compared to the distance-dependent network, the probability-dependent network is less sensitive to population size changes, as *R*_*syn*_ establishes a slower increase as a function of the population size. This observation is likely due to the fact that in the probability-dependent network, a more coherent fraction of shell neurons receive entraining signals from the core, with their location being distributed all over the shell. Shell cells that receive signals from the core in the distance-dependent network, on the other hand, are only distributed within the area close to the core. Therefore, a larger cellular population is required for the probability-dependent network to achieve a similar level of synchronization compared to the distance-dependent network. Compared to an abrupt increase in the core, the degree of synchronization increases more gradually in the shell since its corresponding network receives entraining information indirectly through the core. The standard deviation in *R*_*syn*_ computed from 50 network realizations for each N was relatively large for shell cell numbers smaller than 180. The average degree of synchronization was nearly independent of population size for *N*_*core*_>20 and *N*_*shell*_>300 cells. *R*_*syn*_ values in the two topologies show a similar trend of increase as cell number increases. Experimentally, electrical recordings of the SCN population pattern usually show a near sinusoidal pattern (66). Recordings of varied population sizes revealed that roughly 50 neurons are required to produce the measured population waveform. With fewer neurons in the recording, the pattern is not entirely representative of the actual neuronal distribution within the SCN (11). In accordance with such findings, our results indicate larger cell populations enhance the phase synchronization of the heterogeneous neurons and are necessary for SCN to generate a robust outcome. Therefore, our simulation predictions should not be inadvertently biased when cell populations are sufficiently large.

**Figure 4:**
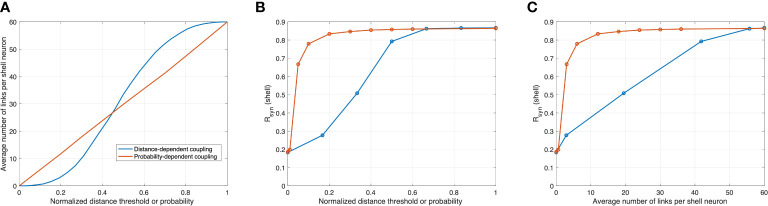
Correlations between synchronization in the shell, normalized distance threshold $\frac{D_{PT}}{D_{PT,max}}$ in the distance-dependent model, probability *P*_*PT*_ in the probability-dependent model, and the average number of links per shell neuron. Orange and blue curves are modeled with distance-dependent and probability-dependent networks, respectively. **(A)** as probability increases, the average connection rises linearly in the probability-dependent network, while as the distance threshold increases, the average connection rises in a sigmoid fashion. **(B)** and **(C)** in the probability-dependent network, the synchronization level increases rapidly as functions of probability and average connections, while this increase is more graduate in the distance-dependent model.

To compare the synchronization efficiency of the two proposed networks, we calculated changes in the average number of connections per shell neuron as a function of two adjustable parameters: normalized distance threshold $\frac{D_{PT}}{D_{PT,max}}$ of the distance-dependent model and probability *P*_*PT*_ of the probability-dependent model. In **Figure 4A**, the average connection number increases monotonically as the distance threshold or probability increases. While the average connection number increases linearly with probability, the average connection number exhibits a sigmoid increase with the distance threshold, affected by the locations of the SCN core and shell neurons. We calculate *R*_*syn*_ under different distance thresholds and probabilities. Compared to the diffusible network, *R*_*syn*_ increases rapidly in the probability-dependent network, as shown in **Figure 4B**. Specifically, an abrupt increase in the degree of synchronization was observed for 0<*p*<0.2 . An asymptotic *R*_*syn*_ value was achieved at *p*=0.4 in the probability-dependent model, while a similar level of synchronization was achieved for a normalized distance of 0.6 in the distance-dependent model. We further plotted *R*_*syn*_ versus average connections per cell for two networks, as shown in **Figure 4C**. *R*_*syn*_ increases rapidly in the probability-dependent network and shows nearly complete synchronization under a relatively low connection number compared to the distance-dependent network. The predicted asymptotic *R*_*syn*_ of 0.86 was close to the value calculated from SCN slice data (67). It should be noted that the lack of full synchronization (*R*_*syn*_=1 ) is due to the cellular heterogeneity of the population rather than the insufficient coupling of the neurotransmitters. Our results indicate that a minimum number of core-shell connections is necessary and sufficient to mediate synchronization in the heterogeneous population in a probability-dependent coupling network.

**Figure 5:**
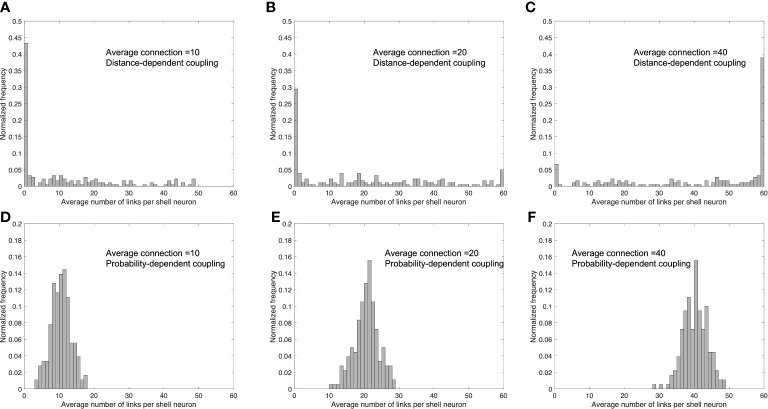
Distribution of the number of links per shell neuron in distance-dependent and probability-dependent networks under different values of the average number of links per shell neuron. Average link number=10 **(A, D)**; average link number=20 **(B, E)**; average link number=40 **(C, F)**. **(A–C)** are simulated under a distance-dependent network, while **(D–F)** were simulated under a probability-dependent network. Compared to the biased distribution in the distance-dependent coupling network, the probability-dependent network adopts a more uniform distribution, resulting in more cells in the shell receiving a moderate number of coupling signals from the core.

To investigate the underlying reason for the probability-dependent network being more efficient in synchronization, we calculated the degree distributions of the connecting numbers with different average connection numbers of two networks, as shown in **Figure 5**. In the diffusible network, the number of connections follows a relatively uniform distribution when the connections are not the highest or the lowest (in our case, the total node number is 60, so the highest connection is 60 while the lowest connection is 0). When the average connection number increases in the distance-dependent network, the highest distribution frequency shifts from the lowest connection (0) to the highest connection (60), with the frequencies in between nearly unchanged. In the probability-dependent network, the stochastic nature of coupling induces a normal distribution, with more cells receiving at least one connection from the core. As the average connection number increases, the distribution of the probability-dependent network shifts toward the right, with the distribution pattern unchanged. Taken together, our findings show that the distribution of connections plays a critical role in a population’s degree of synchronization. Compared to the biased distribution pattern induced from the distance-dependent network, the distribution of the probability-dependent network results in more cells receiving a moderate level of the signal, which leads to a higher synchronization. According to our simulation results, a fundamental advantage of the probability-dependent network is that it promotes synchronization across cell populations with a smaller number of average connection degrees per cell. Therefore, we consider the probability-dependent network a low “energy budget” network (67). To character the SCN network in a more energy-efficient manner, we will focus our discussion on the simulation result obtained by the probability-dependent coupling network.

### Intrinsic synchronization dynamics of the SCN

**Figure 6:**
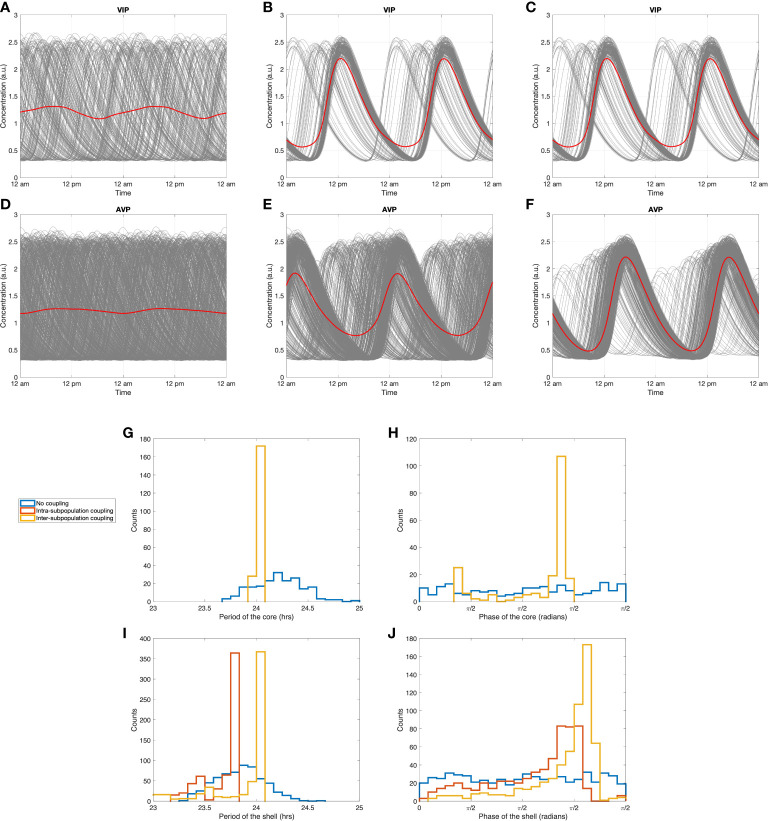
Synchronization dynamics of the SCN during constant darkness. Circadian dynamics of VIP **(A)** without any inter-neuronal coupling, **(B)** with intra-subpopulation coupling, **(C)** with intra-subpopulation and inter-subpopulation coupling (with probability *P*_*PT*_=0.3 ). Circadian dynamics of AVP **(D)** without any inter-neuronal coupling, **(E)** with intra-subpopulation coupling, **(F)** with intra-subpopulation and inter-subpopulation coupling (with probability *P*_*PT*_=0.3 ). **(G, I)**: period distributions of the core and the shell under three coupling conditions. **(H, J)**: phase distribution of the core and the shell under three coupling conditions. Blue curves: distributions without any inter-neuronal coupling. Red curves: distributions with intra-subpopulation coupling. Yellow curves: distributions with both intra-subpopulation and inter-subpopulation coupling.

The existence of neuronal communication within and across the two subpopulations of the SCN is an important and novel aspect of the model. Understanding the system’s inherent synchronization processes is crucial to uncovering underlying physiological relationships. Since at the ensemble level, VIP and AVP, secreted by the core and the shell respectively, serve as the SCN’s output that entrains the downstream compartments, we use VIP and AVP signals as our representative variables for the core and the shell compartments. To understand the stochastic nature of the system independent of entrainment, we investigated the distribution of single-cell phases and periods of the core and the shell in the absence of coupling. In **Figures 6A, D**, we examine single-cell phase distribution when the coupling strengths (*v*_*c*1_ , *v*_*c*2_ , *k*_*c*_ in Equations 2 and 10) are set to 0 for all cells. In both core and shell, the clocks maintain their oscillatory characteristics displaying a broad distribution of periods and phases with the degree of synchronization values close to zero (**Supplementary Table 2**), indicating little to no synchrony among the SCN neurons. Cell phases in both the core and shell are uniformly distributed through the entire regime from 0 to 2*π* (blue curves in **Figures 6H, J** **)**. In terms of periods, the stochastic dynamics of individual neurons produce a normally distributed pattern of values with a mean period of 24.21 *h* for the core and 23.84 *h* for the shell (blue curves in **Figures 6G, I**). Because of this asynchrony, dampened, low-amplitude ensemble average profiles for VIP and AVP are observed. This result indicates that the stochasticity introduced by sampling leads the individual cells to adopt random phases and periods, thus exhibiting a damped ensemble AVP and VIP averages in the absence of an entraining signal.

To explore the entraining impact of the intra-subpopulation coupling induced by neurotransmitters, simulations were performed with the non-zero values for inter-neuronal coupling strengths *v*_*c*1_ and *v*_*c*2_ . In **Figures 6B, E**, the intra-subpopulation communication within the core and the shell induced by VIP and AVP signals significantly augmented the entrainment level indicated by higher *R*_*syn*_ values of VIP and AVP (**Supplementary Table 2**). The distributions of phase and amplitude were more concentrated, with an intrinsic period of 24.05 *h* for the core and 23.69 *h* for the shell (red curves in **Figures 6G–J**). The slightly shorter (1.5*%* ) intrinsic period of the shell than that of the core is in accordance with experimental findings (68).

**Figures 6C, F** investigated the synchronization effects of inter-subpopulation (core-shell) couplings. The coefficient *k*_*c*_ , which quantifies the VIP coupling force of the shell, is set to its non-zero value, imposing entrainment of VIP secreted by the core on the shell subpopulation. VIP affects the shell ensemble in two ways: (1) it induces a more robust pattern with a higher ensemble amplitude (compare **Figures 6E, F**); (2) it imposes the period of the core onto the shell clocks so that a larger amount of the shell population shares the same period with the core (compare the red and yellow curves in **Figure 6I**). As a result, the standard deviation of cell phases in the shell gets smaller than in the case when the inter-subpopulation coupling forces are absent (**Figure 6J**), indicating that the shell maintains a higher degree of synchronization with projections from the core. Taken together, these results provide further evidence that the connections between the core and the shell in the probability-dependent model promote synchronization among sparsely connected shell populations in the absence of light. Our simulations suggest that the observation that separating the dorsal and ventral SCN abolishes synchrony in the dorsal SCN (12) could be related to the role of core-shell connections in increasing the synchronization of the shell oscillators, possibly by selectively cutting VIP connections projecting from the ventral SCN to the dorsal SCN.

**Figure 7:**
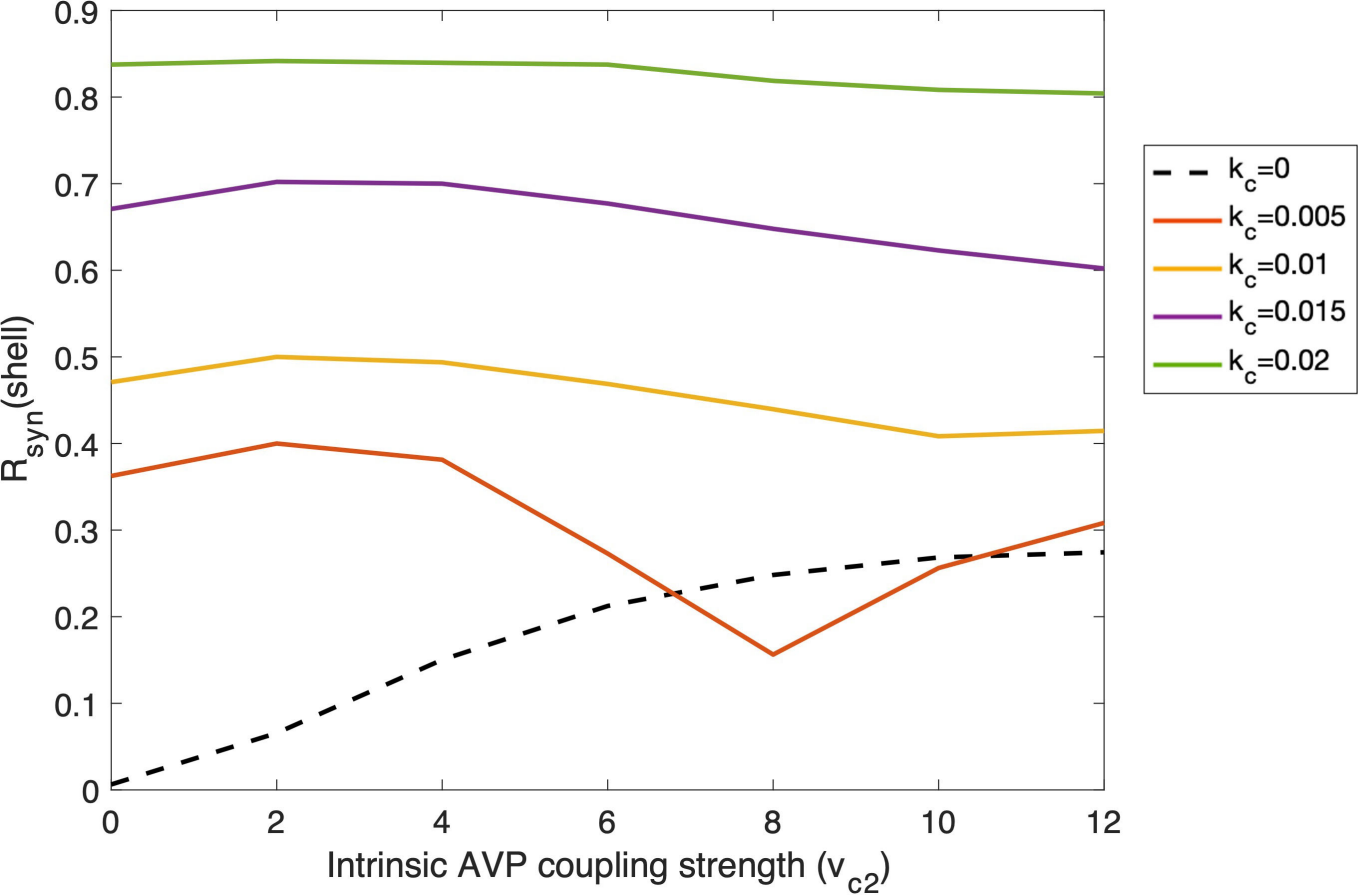
Changes of degree of synchronization within the shell as functions of intra-subpopulation coupling strength of AVP (*v*_*c*2_ ) under the entrainment of VIP signals with different external coupling strength (*k*_*c*_ ). Simulation was conducted with 50 cells in the core and 150 cells in the shell. The probability of the coupling between the core and the shell is 0.5. Dashed line: the shell ensemble only receives AVP coupling signals; Solid lines: the shell ensemble receives both AVP and VIP coupling signals, with VIP coupling strength varying.

To better understand the interplay between intra- and inter-subpopulation coupling, we tested how *R*_*syn*_ in the shell varies as a function of its intrinsic coupling strength both with and without the external VIP signals. As shown in **Figure 7**, when AVP (i.e., *v*_*c*2_ ) is the only coupling force of the shell ensemble (i.e., *k*_*c*_=0 ), *R*_*syn*_ increases as the AVP coupling strength increases, reflecting an increase in the robustness of the intrinsic shell ensemble. We calculated the synchronization degree when the internal and external coupling forces both exist in the system, with different VIP inter-subpopulation coupling strengths. When the VIP coupling strength is relatively high (*k*_*c*_ equals four times its nominal value), the synchronization of the system is almost complete; thereby, no significant change was observed in *R*_*syn*_ as the AVP coupling strength increases. When the VIP coupling strength is moderate (*k*_*c*_ equals two-three times its nominal value), an increase followed by a decrease in *R*_*syn*_ was observed as AVP coupling strength rises. At the beginning stage, a weak intrinsic coupling facilitates the system to get entrained by an external signal. However, as the intrinsic coupling strength grows, it can become a resistant force that contradicts the external coupling strength. When the VIP coupling strength is relatively weak (*k*_*c*_ equals its nominal value), we observed another increase in *R*_*syn*_ following its previous increase and decrease phases, indicating that the intrinsic coupling became the principal driving force. Therefore, our results reveal an antagonistic correlation between the intrinsic and external coupling forces. Circadian timing systems are both robust to external changes in the environment and plastic to adapt to environmental changes such as temperature and nutrient conditions (69). Our results reflect a complex relationship between the robustness and flexibility of the multi-cellular oscillatory system. More details regarding this trade-off will be included in “Discussion”.

### Light entrainment of the system under different photoperiods

**Figure 8:**
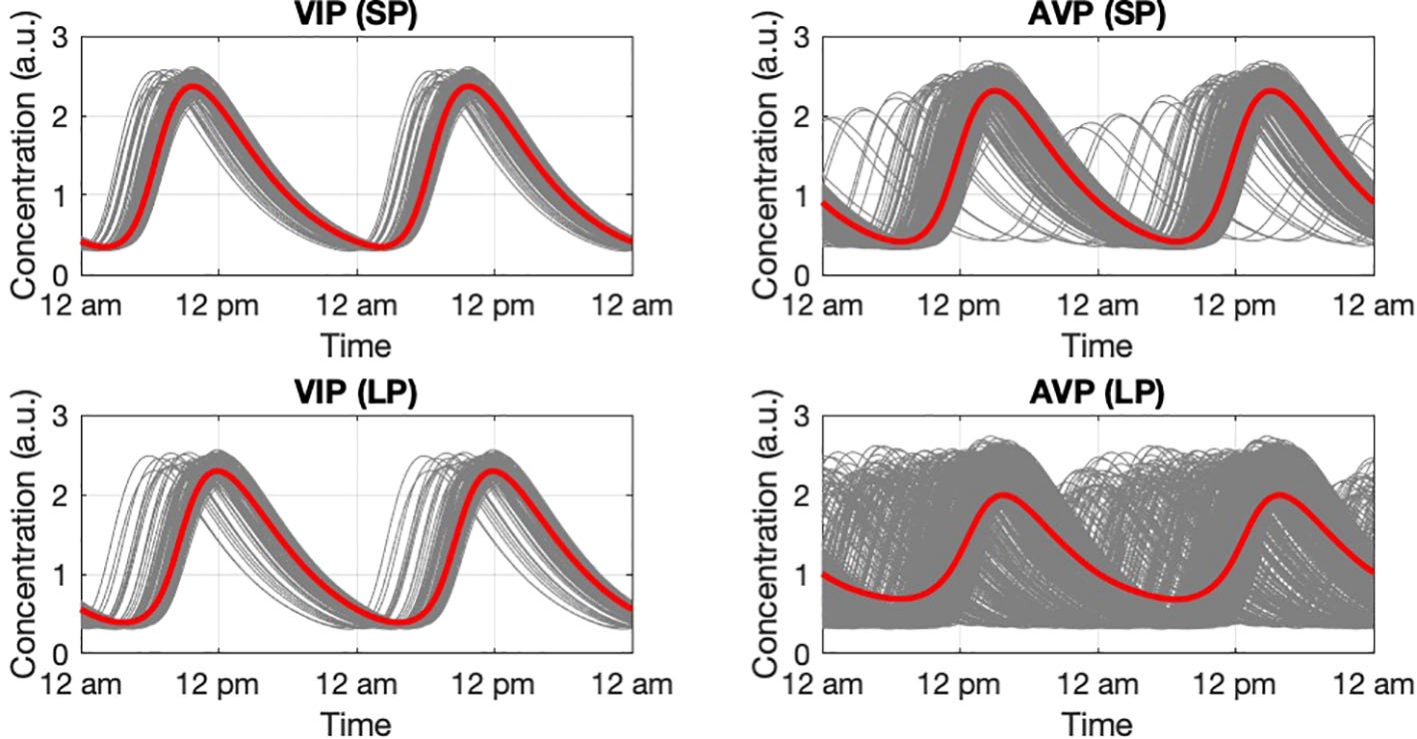
Circadian dynamic of the core and shell under short photoperiod and long photoperiod. VIP and AVP circadian profiles were plotted as they are the representative components in the core and the shell. During SP, both the core and the shell have a narrower phase distribution compared to LP. The higher synchronization level results in an increase in the ensemble average amplitude. The simulation was conducted with 200 cells in the core and 600 cells in the shell.

We further tested the response of the SCN when entrained by different photoperiods (PP) in a way mimicking seasonality (54, 70). **Figure 8** depicts the photoperiod-dependent circadian profiles for the SCN core and shell under LP (16L/8D) and SP (8L/16D). Entrained by L/D cycles, the synchronization of the SCN is significantly increased with the ensemble oscillations of both the core and the shell adopting periods (~ 24 *h* ) equivalent to the external photoperiod. Under LP, both the core and the shell ensembles showed smaller degrees of synchronization compared to that of SP, indicated by the broader phase distributions of VIP and AVP profiles. Therefore, the model consistently identified a greater asynchrony level under LP, which agrees with the experimental findings (71).

**Figure 9:**
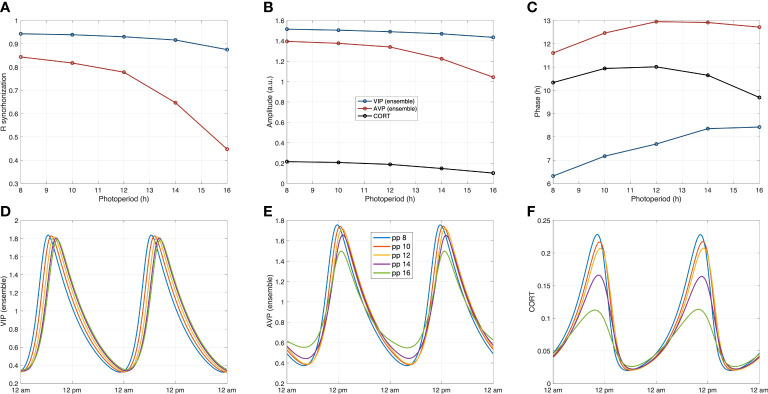
System’s dynamics under photoperiod changes. **(A)** The calculated synchronization level Rsyn in both the core and the shell decrease as the photoperiod increases. **(B)** The reduced synchronization results in lower ensemble average amplitudes of the core and the shell under longer photoperiods, which further leads to the decrease in corticosterone amplitude. **(C)** The core exhibits a phase delay as the photoperiod increases, while the phase changes of the shell and the HPA axis are nonmonotonic. **(D–F)**: The circadian dynamics of the representative components of the core the shell, and the HPA axis under different photoperiods. The phase and amplitude relationships are consistent with the observations in Figures **(A–C)**.

To obtain additional insights into the ways photic signals propagate from the core and shell to the HPA under different PP, we examined the circadian profiles of three compartments (core, shell, and HPA axis) as photoperiods gradually shift from SP to LP (*PP* = 8, 10, 12, 14, 16 *h* ). As depicted in **Figure 9**, the amplitude of three representative components, VIP (ensemble), AVP (ensemble), and CORT, decreased as the photoperiod increased. Our model predicts a higher AVP ensemble oscillation inducing higher corticosterone amplitude in SP. Such an increase of CORT in winter (shorter PP) was also reported in both experimental and modeling works (32, 70). Moreover, the peak time of the core exhibits phase delay as photoperiod increases, while that of the shell and the HPA axis firstly exhibit a phase delay and then show slight phase advance, with the most delayed phase achieved at equinoctial *zeitgebers* (*PP* = 12 ). The peak time of CORT during SP is slightly delayed compared to LP. This delay is manifested as the peak phase shift to the right during SP for the CORT oscillations shown in **Figure 9F**. Experimental studies reported a phase advance of cortisol under long-photoperiod summer compared to winter (72). Moreover, previous modeling results also suggested a phase delay occurred in the HPA axis and a phase advance in the SCN populations during SP (9, 70). Taken together, our model predicted glucocorticoid phase shifts under different photoperiods in accordance with previous findings.

### The effect of temporal topological switching of the probability-dependent network

**Figure 10:**
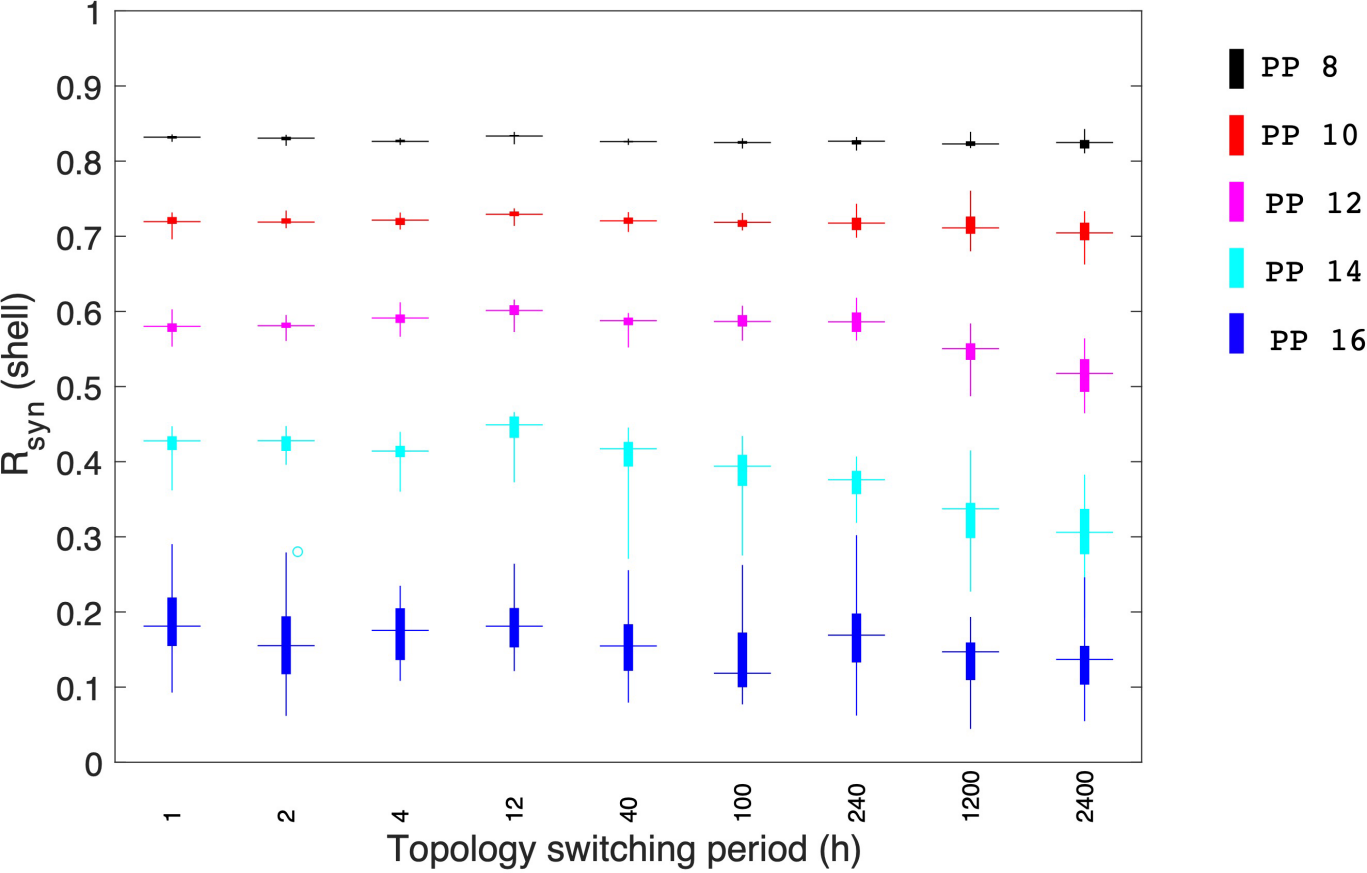
Effects of topologies switching between the core and shell coupling during individual simulations. Synchronization levels were examined under different photoperiods. During short photoperiods (PP 8 and 10), when the synchronization is almost complete, topology switching doesn’t result in any significant effect on the system’s synchrony. When photoperiods are moderate, a fast topology switch can enhance the synchronization level of the core. Under long photoperiods when the synchronization level is relatively low, the topology switch failed to enhance the system’s synchronization. 50 realizations were conducted in our simulation.

Stochastic network reorganization is constantly observed in physiological systems (73), complicating the functional understanding of the SCN since the network changes over time (8). To investigate the interplay between the spatiotemporal architecture reorganization of the SCN and the synchronization of the physiological features of the circadian system in our probability-dependent coupling model, we also considered the possibility of changing topologies during the course of the simulations. We calculated *R*_*syn*_ under different topology switching periods which describe the interval under which the core-shell connection changes (e.g., a 2 h switching period means that the core-shell couplings are reorganized every two hours). **Figure 10** depicts how the degree of synchronization changes as a function of the switching period under different photoperiods. For each switching period under a certain photoperiod, 50 network realizations were calculated to account for statistical network behaviors. For a short photoperiod (*PP*=8 ), 50 realizations of the probability-dependent coupling produced an almost complete degree of synchronization with a slight standard deviation under all the simulated switching periods. This indicated that switching topologies in a highly synchronized system will not significantly affect its synchronization behavior. Moving to larger photoperiods (*PP*=10, 12, 14 ), an overall drop in *R*_*syn*_ and increase in standard deviation was observed as the value of the switching period increases. These results indicate that when the synchronization is not complete, a fast-switching topology process in the SCN probability-dependent network helps achieve a higher degree of synchronization with a minimum connection number. Moreover, as the switching period increases, the synchronization status calculated from different realizations tends to differ more from each other. The synchronization processes in longer photoperiods are less consistent considering the heterogeneities in various network realizations. Under long photoperiod (*PP*=16), when *R*_*syn*_ is the lowest with the highest standard deviation, changing shifting frequencies in the network failed to impose any significant effect on the degree of synchronization. The neural network circuitry and synchronization behavior are currently understudied experimentally, and additional research is required to examine the SCN network thoroughly (73). Our model enabled us to study the spatial-temporal network of the SCN circadian oscillators. The results suggest a potential mechanism for the system to increase its synchronization efficiency. More experimental investigations on this *in silicon* observation are anticipated to be done in the future.

## Discussion

The synchronization of the hierarchical circadian timing system is critical for maintaining homeostasis. The central SCN pacemaker is a heterogeneous organization consisting of different subpopulations classified by their anatomical connections and neurotransmitter identity (17). The entrainment signals from the SCN are conveyed to the periphery through the central effector (HPA axis) that controls organismal behavior. Dysregulation of the system through alterations of the timing of *zeitgebers* (jet lag, shift work, disruptions of feeding time, etc.) can lead to the incidence of a variety of pathologies, including metabolic syndrome, diabetes, obesity, tumorigenesis, and auto-immune disorders (74, 75).

Since the light/dark ratio has been identified as one of the factors that affect the synchronization of circadian entrainment, the complexity of the dynamic response of the circadian timing system under different photic conditions requires a systematic approach to investigate a multitude of interactions. As an extension of our previous work (45), the present study integrated a multi-compartment SCN model with the downstream HPA axis, taking into account the flexible inter-SCN coupling with varying photoperiods. Utilizing the model, we tested the synchronization efficiency of two potential SCN inter-subpopulation coupling mechanisms, the temporal topology changes of the SCN, as well as the circadian dynamics of the HPA axis under different LD ratios.

While the mechanisms underlying interactions between neuronal nodes in the SCN network remain unclear, mathematical modeling can help elaborate on the effects of potential topologies and examine the collective behavior of such complex networks (28). We modeled both distance-dependent and probability-dependent coupling topologies for the SCN to adjust its phase distributions according to photoperiod changes since both diffusible and synaptic signals can contribute to the coupling mechanisms behind the core-shell communication (29). By comparing the two mechanisms, we hypothesize that the probability-dependent network requires fewer connections to achieve the same degree of synchronization compared to the distance-dependent model (**Figures 4**, **5**). Wiring distant neurons across the brain is a costly mechanism that depends on the network coupling volume (76). Considering the “energy budget” (numbers of connections required to generate a certain level of synchronization) of connecting neuronal cells throughout the brain due to the signal transmission (77), probability-dependent coupling networks are therefore predicted to provide more efficient connectivity patterns while maintaining the same performance in terms of rhythm generation and cellular synchronization of the SCN. Our model provides alternative explanations for the distinct synchronization of the SCN neurons under different photoperiods by considering the diffusible distance threshold or the connection probability across the core and the shell (**Figure 2**). Both the distance threshold and connection probability are hypothesized to decrease as the photoperiod increases (Equations 32 and 34). Thus, fewer cells in the shell can receive signals from the core, therefore becoming less synchronized. While the real SCN network is more complex than the proposed models and can include both mechanisms at the same time, our results suggest that the probability-dependent coupling might be more realistic for the SCN network to diminish its energy budget.

By exploring the intrinsic dynamics of the SCN inter-neuronal coupling, our results indicate that autocrine neurotransmitter activation is sufficient to sustain oscillations in the SCN topology (**Figure 6**). The intra-neuronal coupling strength induces a mild increase in *R*_*syn*_ , which agrees with the fact that the behavioral and physiological rhythms regulated by endogenous circadian clocks persist even without environmental cues such as light and food (78). In rodents, the transcription of *Cry1* showed a robust and sustained circadian rhythm without damping circadian amplitude in both LD and DD (79), meaning that the SCN has topographically structured coupling mechanisms and can maintain synchronization even in complete darkness. In accordance with such findings, our model predicts highly synchronized SCN network can be achieved by the coupling effects of its neurotransmitters. This self-sustained synchronization is essential for the coherent circadian output of the SCN to regulate the downstream activities.

Biological systems exhibit both robustness to environmental changes and plasticity to adapt to external conditions (69). By comparing the synchronization degree of the shell under different combinations of inter-subpopulation and intra-subpopulation coupling strengths, we identified a trade-off between robustness/rigidity and plasticity/flexibility of the SCN network induced by the antagonistic effects of the intrinsic and external coupling forces (intrinsic force: AVP intra-subpopulation coupling; external force: VIP inter-subpopulation coupling, **Figure 7**). When the intrinsic or the external force is the principal driver, the degree of synchronization increases as the intrinsic coupling force rises. During the range when the two forces play a relatively equivalent role, the increase of the intrinsic force increases the rigidity of the system, making it more difficult to get entrained by the external signal. This indicates that the SCN oscillator must be strong enough to withstand external noise while also being flexible enough to perform as an entrainable clock under varying photoperiods (e.g. seasons). Experiments reveal that compared to oscillators in the periphery, the intercellular communication among SCN cells through the neuronal and diffusible networks is the unique feature of the SCN (2). On the other hand, almost no coupling exists in the periphery, which ensures that they stay flexible when responding to signals from the SCN (80). Therefore, by strengthening its intrinsic coupling strength, the SCN clock developed rigidity to act as a noise filter to the external entraining signals.

By modifying the network structure in terms of inter-subpopulation neuronal connections, we modeled the system’s response to photoperiod changes. Our results show a non-monotonical pattern for phases in the shell and HPA axis to shift as the photoperiod progresses from LP to SP (**Figure 9**). Therefore, our model hypothesizes a strategy where the response of the HPA axis under different photoperiods is regulated by the distribution of the AVP ensemble outcome. In line with average ensemble amplitudes, *R*_*syn*_ metrics of the core and shell populations further testify to the seasonal differences in synchrony, with PP 8 exhibiting the most synchronous oscillations and PP 16 the most asynchronous. Interestingly, as the photoperiod increases, synchronization levels in the shell first show a slight decrease and exhibit an abrupt drop between PP 12 and 16, indicating that the spring, autumn, and winter seasons are more synchronized than the summer season, in accordance with results reported previously (54).

Consequently, the higher degree of synchronization of the shell under short photoperiods results in a higher level of CORT. Seasonal changes can cause misalignment of the physiological systems. For instance, seasonal affective disorder (SAD) is characterized by a cyclical pattern of depression that occurs during short photoperiods and then fades during longer photoperiods (81, 82). Different individuals react differently to seasonal changes and can develop differential severity and symptoms of SAD. For example, a fraction of SAD patients exhibits a variety of secondary symptoms, such as sleep disturbances, lethargy, and carbohydrate cravings, while others are less sensitive to seasonal changes (83). SAD also affects men and women in different ways, with women three to five times more likely than males to be affected (84). We anticipate that these individualized differences can be accounted for by the parameters in Equations 32 and 34, which affect the plasticity of the system during seasonal changes. The plasticity of their SCN networks is hypothesized to be lower in people who are more resilient to seasonal variations. Further theoretical simulations regarding this will be conducted in the future.

Studies show that individual SCN neuron dynamics are not static. For example, wild-type SCN neurons can randomly switch from rhythmic to nonrhythmic and vice versa (2). Furthermore, even mutant SCN neurons occasionally display infrequent, intermittent PER2 oscillations, suggesting that rhythmicity is a stochastic event (85). Inspired by the stochastic nature of the circadian oscillators, we hypothesized that the coupling network which connects the core and the shell could change its topology over time. By modeling the temporal topology shifts of the core-shell coupling (**Figure 10**), we predict a positive correlation between the topology switching speed and the degree of synchronization when the synchronization is not complete. The fundamental rationale might be that by changing the inter-subpopulation topology quickly, more cells in the shell will be able to receive signals from the core over time. Therefore, our model suggests a potential mechanism for the SCN to achieve a higher degree of synchronization when the probability-dependent connection numbers between two subpopulations are insufficient.

In summary, mathematical modeling can provide substantial insight into physiological systems (86, 87). Our model attempts to provide explanations of the variations in SCN and HPA axis responses as photoperiod changes. While testing two possible topologies for the SCN core-shell coupling networks, the probability-dependent network was suggested as a low “energy budget” architecture. We also identified the antagonistic effects of inter-subpopulation and intra-subpopulation coupling on the SCN network’s resilience and stiffness. Moreover, the temporal topology switch of the network has been predicted to facilitate multi-cellular synchronization for the first time. Since our model incorporates the essential hierarchical structure of the SCN, the HPA axis, and their response under different photoperiods, it should be a valuable tool for multiple additional studies, including the investigation of the individualized seasonal response of diseases; the inclusion of the downstream entraining of peripheral clocks; and the simulation of seasonal jetlag.

## Data availability statement

The original contributions presented in the study are included in the article/**Supplementary Material**. Further inquiries can be directed to the corresponding author.

## Author contributions

YL developed the model, designed and conducted the calculations, analyzed the results, and prepared the manuscript. IA conceived the study, analyzed the results, edited manuscript. All authors contributed to the article and approved the submitted version.

## Funding

The authors acknowledge financial support from NIH GM131800.

## Conflict of interest

The authors declare that the research was conducted in the absence of any commercial or financial relationships that could be construed as a potential conflict of interest.

## Publisher’s note

All claims expressed in this article are solely those of the authors and do not necessarily represent those of their affiliated organizations, or those of the publisher, the editors and the reviewers. Any product that may be evaluated in this article, or claim that may be made by its manufacturer, is not guaranteed or endorsed by the publisher.
